# Telemedicine in the primary care of older adults: a systematic mixed studies review

**DOI:** 10.1186/s12875-023-02085-7

**Published:** 2023-07-20

**Authors:** Marwa Ilali, Mélanie Le Berre, Isabelle Vedel, Vladimir Khanassov

**Affiliations:** 1 Department of Family Medicine, McGill University, 5858 Côte-Des-Neiges Road, 3Rd Floor, Montreal, Québec H3S 1Z1 Canada; 2 School of Rehabilitation Science, Université de Montréal, Montreal, Canada; 3 Research Center, Institut Universitaire de Gériatrie de Montréal, Montreal, Canada; 4 Goldman Herzl Family Practice Centre, Jewish General Hospital, Montreal, Canada

**Keywords:** Telemedicine, Family Practice, Primary Care, Aged, Systematic Review, Mixed Methods

## Abstract

**Background:**

Family physicians had to deliver care remotely during the COVID-19 pandemic. Their efforts highlighted the importance of developing a primary care telemedicine (TM) model. TM has the potential to provide a high-quality option for primary care delivery. However, it poses unique challenges for older adults. Our aim was therefore to explore the effects of TM and the determinants of its use in primary care for older adults.

**Methods:**

In this systematic mixed studies review, MEDLINE, PsycINFO, EMBASE, CINHAL, AgeLine, DARE, Cochrane Library, and clinical trials research registers were searched for articles in English, French or Russian. Two reviewers performed study selection, data extraction and assessment of study quality. TM’s effects were reported through the tabulation of key variables. TM use determinants were interpreted using thematic analysis based on Chang’s framework. All data were integrated using a joint display matrix.

**Results:**

From 3,328 references identified, 20 studies were included. They used either phone (*n* = 8), videoconference (*n* = 9) or both (*n* = 3). Among studies reporting positive outcomes in TM experience, ‘user habit or preferences’ was the most cited barrier and ‘location and travel time’ was the most cited facilitator. Only one study reported negative outcomes in TM experience and reported ‘comfort with patient communication’ and ‘user interface, intended use or usability’ as barriers, and ‘technology skills and knowledge’ and ‘location and travel time’ as facilitators.

Among studies reporting positive outcomes in service use and usability, no barrier or facilitator was cited more than once. Only one study reported a positive outcome in health-related and behavioural outcomes.

**Conclusions:**

TM in older adults’ primary care generally led to positive experiences, high satisfaction and generated an interest towards alternative healthcare delivery model. Future research should explore its efficacy on clinical, health-related and healthcare services use.

**Supplementary Information:**

The online version contains supplementary material available at 10.1186/s12875-023-02085-7.

## Key points

- Most health systems have been able to adapt quickly to virtual consultations with their patients, but there are still opportunities for improvement for older populations.
- Older patients want to have access to both in-person and virtual consultations when appropriate.
- Telemedicine for older adults’ primary care generally led to positive experiences, high satisfaction towards alternative healthcare delivery model.

**Supplementary Information:**

The online version contains supplementary material available at 10.1186/s12875-023-02085-7.

## Impact statement

We certify that this work is novel of recent novel clinical research. This mixed studies review provides insightful findings on the effects of telemedicine on the general care experience, the service use and usability and the health-related and behavioural outcomes of older adults, in addition to uncovering the determinants of its use by this population. Its conclusions can guide primary care clinicians in an optimal use of telemedicine by listing key elements to foster a clinical context favourable to telemedicine use with an older population.

## Why does this paper matter?

This review was designed to explore the literature to understand telemedicine in primary care for older adults. The assessment of the impact of on the general care experience, service use, and health-related outcomes of older adults, as well as determinants of telemedicine use, will inform the qualitative descriptive study of a larger multi-phase research.

## Introduction

The COVID-19 crisis has substantially changed the delivery of primary care. Indeed, with the public health measures, a lot of clinic-based care turned into virtual remote care [1]. Telemedicine (TM) became pervasive. TM refers to an alternative to in-person clinic-based care, and is defined as synchronous remote teleconsultations using phone or video [2]. Prior to the pandemic, research already suggested that TM was an effective approach to deliver medical care, including for older adults [3]. Post-pandemic reflections now suggest it could definitely constitute an asset, not only as part of an emergency response but as mainstream usual primary care by providing remote triage, routine follow-up, and remote care [4].

Yet, due to potential age-related changes in perceptual, motor, or cognitive capacities, older adults may present different needs from the general population, potentially affecting the impact of TM among this population and even their use of the approach [5]. Also, most of the available evidence on TM generally focuses on younger populations [6, 7]. As many experts debated on the extent to which COVID-19-related adaptations will be maintained post-pandemic [8], exploring the potential of TM for the primary care of older adults is essential.

Therefore, this systematic mixed studies review aimed to: 1) To assess the effects of TM on the general care experience, service use, as well as on health-related outcomes in a context of primary care practice for older people, 2) To explore the determinants of TM use in the primary care practice of older people.

## Methods

This systematic mixed studies review followed the Preferred Reporting Items for Systematic Reviews and Meta-Analyses (PRISMA) statement [9]. We included studies with a variety of designs, either quantitative, qualitative or mixed-methods [10]. Mixed studies reviews are appropriate to understand and conceptualize multi-dimensional complex phenomena [11, 12]. This review is a first phase of a multiphase study on telemedicine for older adults in primary care [13]. The review protocol has been recorded at the PROSPERO, CRD42021237686 https://www.crd.york.ac.uk/prospero.

### Data sources

The key concepts of ‘telemedicine’, ‘aged’, and ‘primary health care’ were combined using Boolean logic [14], also using additional related terms such as “ “Video consult*”, “Remote consultation”, “Distance counseling”, “Virtual consultation”. A complete list of terms used for the EMBASE search strategy is available in Supplementary Text S1.

A systematic search was performed by a specialized librarian in MEDLINE, PsycINFO, EMBASE, CINAHL, AgeLine, the Database of Abstracts or Reviews of Effects (DARE), the Cochrane Library (Cochrane Database of Systematic Reviews, Cochrane Central Register of Controlled Trials (CENTRAL), clinical trials research registers (ClinicalTrials.gov and the WHO’s International Clinical Trials Registry Platform) to identify publications in English, French or Russian, based on our team's linguistic proficiency, published before July 2021. We then conducted a snowballing manual search of the reference lists of the included studies to identify additional relevant papers. A final research update was completed in September 2021.

### Study selection

We included all studies presenting primary findings on TM in a context of primary care for community-dwelling older adults or their caregiver. Adults aged over 65 years old living in the community, caregivers, or healthcare providers involved in older adults’ care were included. Telemedicine was defined as synchronous telecommunication (phone, videoconference), provided by a primary care practice involving a family physician, a nurse, or any other healthcare allied professional of the clinic. Only studies reporting relevant outcomes were included, related to experience, effects, determinants and other outcomes such as satisfaction, users’ experience, intention to use, expectations, and frequency of emergency department visits. Detailed inclusion and exclusion criteria are available in Supplementary Table S2.

Two independent reviewers (MI, MLB) selected articles through a two-step process (titles/abstracts, full-text). Any disagreements were resolved by consensus or the involvement of an additional reviewer (VK). Companion articles of included studies were examined and treated as one study.

### Data collection

Two reviewers (MI, MLB) independently extracted data using a standardized data collection form. They screened all included articles for: a) study characteristics, including authors, year of publication, country of origin, and study design; b) description of the participants, including sample size, sex, age, and description of the setting of the family medicine practice (e.g., solo vs team-based, healthcare professionals), c) type of TM described and its components (e.g., phone vs video conference), d) any reported outcomes on the experience with TM (e.g., satisfaction with care), health care services use (e.g., number of clinical visits), or clinical outcomes (e.g., health status), e) barriers and facilitators to TM use.

### Data synthesis

**Fig. 1:**
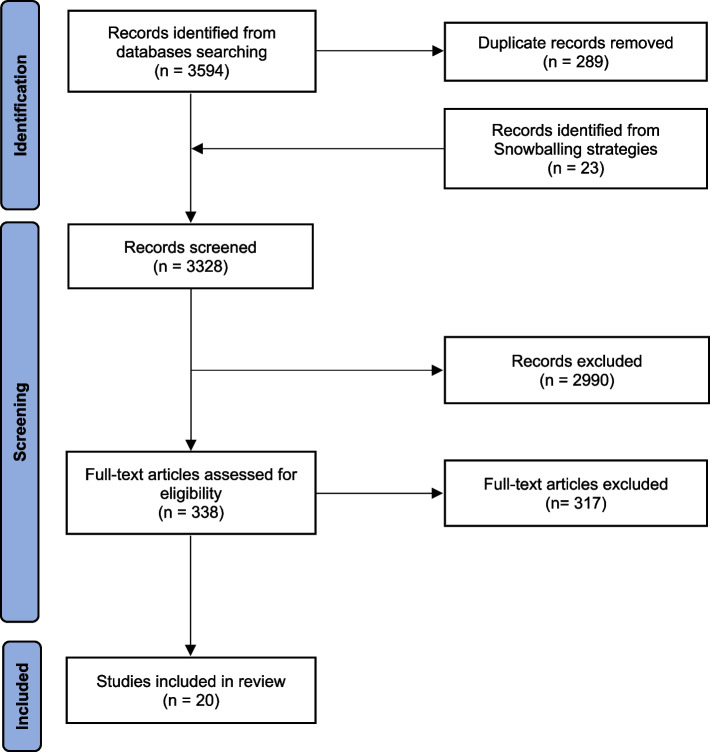
Preferred Reporting Items for Systematic Reviews and Meta- Analyses (PRISMA) flow diagram

This mixed studies review used a parallel-results convergent synthesis design [15] in a three-step process (Fig. 1): (1) analysis of data from quantitative and mixed-methods studies, (2) thematic analysis of data from qualitative and mixed-methods studies, and (3) integration of both findings.

At step 1, we reported TM effects, through findings on the general care experience, on service use and usability and on health-related and behavioural outcomes by tabulating the reported key variables.

At step 2, we coded and interpreted the different reported determinants of TM use using a qualitative thematic deductive analysis based on Chang’s logical framework [16]. Chang’s framework consists of 38 determinants in total, classified into six constructs (healthcare providers, patients, organization, technology, society, and rules/policy), distributed among three dimensions (human, system, and environment). We coded each determinant, identified from the included studies, as either a barrier, an ambivalent determinant or a facilitator.

At step 3, both findings from the previous steps were integrated using a joint display matrix [17]. We combined the findings on TM effects in rows with the findings on determinants of TM use in columns. Two independent reviewers (MI, MLB) visually analyzed patterns and iteratively explored similarities and differences in the direction of findings, in relation to the identified determinants. Any disagreements were resolved by consensus or the involvement of an additional reviewer (VK).

### Quality assessment

Two reviewers (MI, MLB) independently assessed quality of each included study using the mixed methods appraisal tool (MMAT) [18]. The MMAT is a validated critical appraisal tool designed to appraise the methodological quality of qualitative, quantitative and mixed-methods studies. In accordance with MMAT standards, no overall quality score was calculated; studies were appraised as having low, moderate or high methodological quality. Any disagreements were resolved by consensus or the involvement of an additional reviewer (VK).

## Results

### Characteristics of included studies

**Fig. 2:**
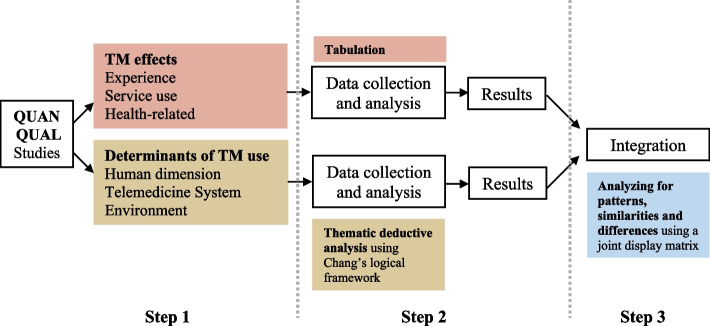
Three-step process of the mixed studies review using a parallel- results convergent synthesis design

The searches initially identified 3,328 references. Of these, 2,990 were excluded based on their title/abstracts and 317 based on their full-text. A total of 20 articles were included in the review (Fig. 2): 11 quantitative [19–29], six qualitative [30–35], and 3 mixed-method studies [36–38]. Their characteristics are summarized in Tables 1 and 2. Overall, the geographic locations of the studies were diverse, with six studies in the United States of America [19, 21–24, 33], two in the United Kingdom [25, 34], two in the Netherlands [20, 36], two in Sweden [31, 38], one in Spain [29], one in Scotland [37], one in Ireland [27], one in Switzerland [35], one in Poland [28], one in Portugal [26], one in China [32], and one in New Zealand [30]. Eight studies focused on TM with phones [19, 21, 23, 25, 26, 30, 34, 35], nine on TM with videoconference [20, 22, 24, 27, 32, 33, 36–38] and three on TM with both phone and videoconference [28, 29, 31]. Overall, studies reported on multiple determinants of TM use in all three dimensions of Chang’s framework [16]. Most cited determinants belonged to the human dimension. The environmental dimension determinants were the less cited. All details on the reported determinants are available in Table 2).

**Table 1:** Characteristics of included studies

**1**^**st**^** author (Year), Country**	**Population (Sample size, main eligibility criteria, age, sex)**	**Setting**	**Types of TM**	**Category of Reported Outcomes **(Measuring tools)
**Randomized Controlled Clinical Trials**				
**Chae (2001) ** [27]**, Ireland**	50 older adults who received home care services, Mean age of 67.5 years old (44% Female)	Home health services (HHS) by the Kwachun Community Health Promotion Center (CHPC)	Videoconference consultation	General care experience (Non-validated questionnaire)
				Service use and usability (Non-validated questionnaire)
**Welch (2000) ** [19]**, USA**	512 older adults visiting their physician, Mean age of 68 years old (0% Female)	Two Veterans' Administration general medical clinics	Phone consultation	General care experience (Validated questionnaire: Patient Satisfaction questionnaire [39])
				Service use and usability (Administrative database—Veterans' Administration (VA) national database)
				Health-related and behavioural outcomes (Validated questionnaire: Short Form-36 Health Survey [40]; Non-validated questionnaire from original telephone care study [41])
**Quantitative Non-Randomized Studies**				
**Benaque (2020) ** [29]**, Spain**	7382 older adults with cognitive disorders, Mean age not reported, (Sex distribution not reported)	Fundacio ´ ACE non-profit organization "memory clinic"	Videoconference and phone consultation	General care experience (Validated questionnaire: Healthcare Professionals’ Perceptions [42])
				Service use and usability (Administrative data)
**Jiwa (2005) ** [25]**, UK**	182 older adults who received a phone consultation with a physician or a nurse, Mean age of 78.5 years old (43% Female)	Five primary care practices	Phone consultation	General care experience (Non-validated Questionnaire)
				Health-related and behavioural outcomes (Validated questionnaire: Patient Enablement Instrument [43])
**Khoong (2020) ** [24]**, USA**	40 older adults who were scheduled to receive a phone consultation with a clinician, Mean age not reported (75.2% Female)	Women’s health or general medicine clinic in an urban safety-net system	Videoconference consultation	General care experience (Non-validated questionnaire)
**Lam (2020) ** [23]**, USA**	4525 older adults from a nationally representative sample of Medicare beneficiaries, Mean age of 79.6 years old (57% Female)	National Health and Aging Trends Study database	Phone consultation	General care experience (Survey data from the National Health and Aging Trend Study [44])
**Van Houwelingen (2015) ** [20]**, Netherlands**	207 nurses providing care to older adults, Mean age not reported (94.5% Female)	Home care organizations located in the middle, western, and southern areas of the Netherlands	Videoconference consultation	General care experience (Non-validated Questionnaire; Validated questionnaire: Positive and Negative Affect Schedule (PANAS) scale [45])
**Quantitative Descriptive Studies**				
**Bujnowska-Fedak (2014) ** [28]**, Poland**	286 older patients visiting their physician, Mean age of 73.8 years old (64% Female)	250 general practices in southwest Poland’s Lower Silesia Province	Videoconference and phone consultation	General care experience (Interviews)
**Jácome (2018) ** [26]**, Portugal**	120,269 older adults calling a nurse-led telephone line, Mean age of 77.3 years old (63% Female)	Nurse-led health triage, screening, counselling and referral telephone line	Phone consultation	Service use and usability (Administrative database—Linha Saúde 24 (S24) administration system—public–private partnership integrated into the National Health Service)
**Samples (2019) ** [22]**, USA**	49 clinicians using telemedicine with older adults, Mean age not reported (Sex distribution not reported)	Seattle Veterans Affairs Primary Care Clinic	Videoconference consultation	General care experience (Non-validated questionnaire)
**Townsend (2001) ** [21]**, USA**	530 older adults who received a phone consultation with a nurse, Mean age not reported (Sex distribution not reported)	3 Veterans' Administration system (electronic record) primary care geriatric Clinic	Phone consultation	Service use and usability (Administrative data, Veterans' Administration system (VA))
**Qualitative Studies**				
**Blozik (2012) ** [35]**, Switzerland**	1 older adult who received a phone consultation with a physician, Age of 69 years old (Male)	Swiss Center for Telemedicine	Phone consultation	General care experience (Case report)
**Foster (2001) ** [34]**, UK**	30 older adults who attended community or day centers, Mean age not reported (87% Female)	Out-of-hours primary care services from community groups based in southeast London	Phone consultation	General care experience (Focus groups)
**Franzosa (2021) ** [33]**, USA**	13 healthcare providers providing care to older adults, Mean age not reported, (86% Female)	Six NYC-area practices in home-based primary care	Videoconference consultation	General care experience (Semi-structured interviews)
				Service use and usability (Semi-structured interviews)
**Kung (2016) ** [32]**, China**	10 older adults who attended a local public primary care clinic, Mean age of 74 years old (60% Female)	Single General Outpatient Clinic (GOPC) in Hong Kong	Videoconference consultation	General care experience (Semi-structured interviews)
**Nymberg (2019) ** [31]**, Sweden**	15 older adults who attended a primary heath care center (PHCC) and had at least one chronic disease (hypertension, diabetes, COPD), Mean age of 73.2 years old (53.3% Female)	Three primary health care centers (PHCCs) in Southern Sweden	Videoconference and phone consultation	General care experience (Focus groups)
**Waterworth (2018) ** [30]**, New Zealand**	21 older adults who attended a general practice and had two or more long-term conditions, Mean age of 77 years old, (52% Female)	Six general Practices involved previously in research in New Zealand	Phone consultation	General care experience (Semi-structured interviews)
				Service use and usability (Semi-structured interviews)
**Mixed Methods Studies**				
**Gabrielsson-Järhult et al. (2021) ** [38]**, Sweden**	10,400 older adults who received a video consultation with a physician, (QUAN) & 26 interviews (QUAL), Mean age not reported, (QUAL: 62% Female)	National study sample registry in a Swedish region	Videoconference consultation	General care experience (Semi-structured interviews)
**Macduff et al. (2001) ** [37]**, Scotland**	173 older adults who received a video consultation with a nurse (QUAN) & 7 interviews (QUAL), Mean age not reported, (Sex distribution not reported)	Town-based general practice	Videoconference consultation	General care experience (Non-validated questionnaire; interviews)
**Van Houwelingen (2018) ** [36]**, Netherlands**	256 older adults from various clubs and organizations (QUAN) & 15 interviews (QUAL), Mean age 70 years old in phase 1 & 87 years old in phase 2, (50% Female in phase 1, 60% Female)	Community Care and general population (Patient advocacy organizations, Senior social clubs, Health care organizations, and a senior information day in Utrecht)	Videoconference consultation	General care experience (Observations; Validated questionnaires: - Technology experience [46] - Older people’s perception [47, 48])
				Service use and usability (Validated questionnaire: Demographic and Health-related [49])

**Table 2:** Summary of barriers and facilitators identified in the included quantitative and qualitative studies, using Chang’s framework

				Barriers	Ambivalent	Facilitators
Dimension	Determinants		Relevant quote from the articles related to the framework dimension	Articles reporting the determinant as a barrier	Articles reporting the determinant as both a facilitator and a barrier	Articles reporting the determinant as a facilitator
**Human Dimension**	**Healthcare Providers**					
	1.1	Comfort with workflow	“Providers were concerned about negative impacts on their clinic flow” [22]	***n* = 1** [22]	***n* = 0**	***n* = 0**
	1.2	Comfort with patient communication	“Many expressed concerns about their needs being assessed over the telephone, including doubts about the ability of unknown doctors to make accurate diagnoses in these circumstances. […] ‘I don’t think it is advisable to talk to the doctor over the phone about what you are suffering with and what the symptoms are and so on. I think it is most important that a doctor sees you’. (Male, group 4.)” [34]	***n* = 5** [21, 30, 33, 34, 37]	***n* = 1** [22]	***n* = 1** [27]
	1.3	Comfort with provider interaction	“There was also the mutually perceived incidental benefit of opportunity for doctor-nurse communication, often carrying over beyond individual cases” [37]	***n* = 0**	***n* = 1** [37]	***n* = 2** [20, 33]
	1.4	Expertise with technology	“Nurses with high technology experience (e.g., computers, microwaves, Skype, tablets) (*n* = 41) had a significantly lower negative affect score related to the use of home telehealth” [36]	***n* = 0**	***n* = 2** [20, 33]	***n* = 2** [22, 36]
	1.5	Education and training	“It was clear that this rather public learning process had been uncomfortable for some of the nurses involved” [37]	***n* = 1** [37]	***n* = 1** [30]	***n* = 0**
	1.6	Resistance to change	“Undoubtedly, this was related to the local team leader’s role in initiating the development, but it was clear that her colleagues also found the development interesting and worthwhile” [37]	***n* = 0**	***n* = 0**	***n* = 3** [20, 22, 37]
	**Patients & Caregivers**					
	2.1	Disease characteristics / sociodemographic characteristics	“Older adults 'described ‘The aging body as a barrier’ with impaired practical abilities such as trembling fingers or impaired vision or hearing” [31]	***n* = 1** [23]	***n* = 13** [21, 22, 26–28, 30–34, 36, 37]	***n* = 0**
	2.2	Technology skills and knowledge	“Older people believed that they were not able to accomplish certain technological tasks (low self-efficacy), but discovered that they actually were able to do so or could do so after a small suggestion on how to proceed” [36]	***n* = 7** [22–24, 28, 32, 33, 36]	***n* = 1** [31]	***n* = 2** [34, 38]
	2.3	User habits/preferences	“’I don’t feel this can work and doesn’t feel real to me. I prefer going to a doctor in a clinic, let the doctor see myself through his own eyes’ “ [32]	***n* = 6** [22, 25, 26, 29, 33, 36]	***n* = 8** [19, 24, 28, 30–32, 34, 37]	***n* = 1** [27]
	2.4	Location/travel time	“One participant felt that e-consultation could break down geographical barriers that potentially reduce access to healthcare […] 'I don't have to waste my time coming in for check-ups'” [32]	***n* = 0**	***n* = 5** [22, 27, 28, 37]	***n* = 7** [21, 29–34]
	2.5	Patient awareness / support	“Although some participants were against e-consultation because of difficulty with internet access, they would consider using the service with the help from family members and friends, as this participant described: ‘It [e-consultation] is only feasible if I can receive help from the younger ones, helping me to use the internet…’ [Fishermen, retired, female, age above 65]” [32]	***n* = 1** [33]	***n* = 1** [32]	***n* = 1** [36]
	2.6	Technology equipment	“26 scheduled visits were cancelled due to lack of patients’ telecommunications devices” [29]	***n* = 6** [24, 28, 29, 32, 33, 36]	***n* = 0**	***n* = 0**
	2.7	Medical cost (out-of-pocket)	“Relying on paid caregivers often meant using the aides’ own phone and data plan. As one provider asked, ‘is someone reimbursing [the aide] for that [data]? Is there Wi-Fi in the home? Do they [the aide] even have a smartphone?’ (CD, Practice 6)” [33]	***n* = 2** [31, 33]	***n* = 3** [20, 30, 32]	***n* = 0**
**System Dimension**	**Organization**					
	3.1	Leadership	“Undoubtedly, this was related to the local team leader’s role in initiating the development” [37]	***n* = 0**	***n* = 0**	***n* = 1** [37]
	3.2	Change management	“While participants generally expressed pride in how quickly they and their patients adapted, they also described limitations […] including the need to rapidly consent patients, set up patient portal accounts, and learn a new system quickly” [33]	***n* = 0**	***n* = 1** [33]	***n* = 0**
	3.3	Budget	N/A	***n* = 0**	***n* = 0**	***n* = 0**
	3.4	Workflow reengineering	“Instead of providing a way to maintain contact with patients without requiring them to appear in clinic frequently, telephone appointments became simply an additional service” [19]	***n* = 3** [21, 30, 31]	***n* = 1** [19]	***n* = 4** [27, 32, 33, 37]
	3.5	Organizational culture	“They also had thoughts about differences between the organizations. One of the participants wondered why short text message reminders are common in the dental care but not in primary care” [31]	***n* = 2** [31, 32]	***n* = 0**	***n* = 0**
	3.6	Hospital information systems	N/A	***n* = 0**	***n* = 0**	***n* = 0**
	3.7	Training and support	N/A	***n* = 0**	***n* = 0**	***n* = 0**
	**Technology**					
	4.1	Reliability of technology	“Some participants demanded high internet stability for the service, as they felt it would be useless if the technology itself was unreliable: ‘If the computer system is slow then it [e-consultation] isn’t helpful. It will take longer if the computer system constantly breaks down and need to spend hours to recover.’” [32]	***n* = 5** [22, 31–33, 36]	***n* = 2** [30, 37]	***n* = 0**
	4.2	Storage	N/A	***n* = 0**	***n* = 0**	***n* = 0**
	4.3	System speed	N/A	***n* = 0**	***n* = 0**	***n* = 0**
	4.4	User interface / intended use /usability	“Overall, providers noted the diversity of options (institutional platforms, other HIPAA-compliant commercial platforms, and consumer platforms) allowed greater access to patients than would otherwise have been possible. Providers appreciated the ease of texting images of a skin condition or meeting quickly by FaceTime and hoped this flexibility could continue as privacy rules were enforced again. ‘We just did whatever we really felt was needed for that patient, and it’s going to be spoiled going back to the regular [institutional platform] way’, noted one social worker” [33]	***n* = 1** [34]	*n* = 9 [21, 27–31, 33, 36, 37]	***n* = 0**
	4.5	Data quality	“GPs [General Practitioners] reported some sound issues, difficulties seeing rashes and skin problems.” [37]	***n* = 1** [37]	***n* = 0**	***n* = 0**
	4.6	Transmission	“Having digital access to information about the medication was described as another potential advantage” [31]	***n* = 0**	***n* = 0**	***n* = 1** [31]
	4.7	Interoperability	“The main issue that the participants talked about was that there was ‘Poor communication between health care organizations’ IT systems’. As no organization was fully updated with all the information, the participants expressed ‘disappointment over poor IT systems’” [31]	***n* = 1** [31]	***n* = 0**	***n *= 1** [33]
	4.8	Information security	““I think e-consultation opens up an opportunity for criminal activities if safety measures [online security] are not taken.’ [Police, retired, male, age above 65]” [32]	***n* = 0**	***n* = 2** [31, 32]	***n* = 0**
**Environment Dimension**	**Society**					
	5.1	3rd party payers	“The accelerated pace of change driven by the pandemic and resulting changes in regulation and reimbursement have also allowed for rapid HBPC practice innovations that would not otherwise have been possible.” [33]	***n* = 0**	***n* = 0**	***n* = 1** [33]
	5.2	Technology infrastructure	N/A	***n* = 0**	***n* = 0**	***n* = 0**
	5.3	Reimbursement	N/A	***n* = 0**	***n* = 0**	***n* = 0**
	5.4	Insurance fee schedule	N/A	***n* = 0**	***n *= 0**	***n* = 0**
	5.5	Social norms and values / temporal trends	N/A	***n* = 0**	***n* = 0**	***n* = 0**
	**Rules/Policy**					
	6.1	Medical liability	N/A	***n* = 0**	***n* = 0**	***n* = 0**
	6.2	Practice certification and license	N/A	***n* = 0**	***n* = 0**	***n* = 0**
	6.3	Governmental authority	“Governmental regulations in the establishment and running of e-consultation services would enhance participants’ trust in the service.’As long as the government is at the back of the service [e-consultation], I would then have confidence in it.’ [Domestic helper, retired, female, age 40–65]” [32]	***n* = 0**	***n* = 0**	***n* = 1** [32]
	6.4	Privacy and security rules	“Providers appreciated the ease of texting images of a skin condition or meeting quickly by FaceTime and hoped this flexibility could continue as privacy rules were enforced again. ‘We just did whatever we really felt was needed for that patient, and it’s going to be spoiled going back to the regular [institutional platform] way’, noted one social worker” [33]	***n* = 0**	***n* = 1** [33]	***n* = 0**
	6.5	Interface standards	N/A	***n* = 0**	***n* = 0**	***n* = 0**

### TM effects on general care experience

Among the 18 different studies reporting on the effects of TM on the general care experience, eleven described the general experience of TM itself, using semi-structured interviews, non-validated questionnaires, focus groups, validated questionnaires or a case report method. Three reported on satisfaction with TM, using a validated questionnaire, a non-validated questionnaire or a combination of a non-validated questionnaire with interviews. Three reported on TM readiness, using longitudinal data from a national survey, a non-validated questionnaire or interviews. One reported on attitudes regarding TM, using both a validated and a non-validated questionnaire. One reported on interest towards TM, using a non-validated questionnaire. One reported on TM acceptability using semi-structured interviews.

Among studies reporting positive outcomes on the general care experience (*n* = 11) [19, 20, 22, 24, 25, 27, 29–31, 37, 38], ‘user habit or preferences’ was the most cited determinant, reported as a barrier by three studies [22, 25, 29], as a facilitator by one study [27] and as a more ambivalent factor by five studies **(**Supplementary Table S4**)** [19, 24, 30, 31, 37]. ‘User habits or preferences’ was also the most cited barrier. Many older adults expressed how they preferred face-to-face interactions and remain within familiar territory, while some mentioned positive experiences with the technology, on which they were ready to build to learn about TM. The most cited facilitator was the ‘location and travel time’, reported as a facilitator by three studies [29–31]. Most saw in TM an increased accessibility, particularly for rural areas, and convenient time saving from both sides.

Only one study reported negative outcomes [34], and reported ‘comfort with patient communication’ as a barrier. It reflected a worry expressed by older patients, who questioned whether primary care clinicians would really be in a position to assess their needs over the phone and ‘make accurate diagnoses in these circumstances’, particularly in the case of a new clinical encounter. In addition, the study also reported ‘User interface, intended use or usability’ as a barrier. Older patients also expressed difficulties with some aspects of TM, notably the use of pre-recorded vocal messages, as useful information was “sometimes given too quickly to be noted down”. In contrast, this study reported both the ‘technology skills and knowledge’ and the ‘location and travel time’ as facilitators. While other studies mentioned distrust, inexperience, unreadiness or lack of self-efficacy with the technology, some older adults even claiming they were ‘digital illiterate’, participants from Foster et al.’s study (2001) [34] reported that they felt confident to use the telephone for “medication queries and minor problems”.

### TM effects on healthcare service use and usability

Among the nine different studies reporting on service use and usability, seven reported on usability, using an administrative database, semi-structured interviews or a validated questionnaire and two reported on the number of clinical visits, using an administrative database or a non-validated questionnaire.

Among studies reporting positive outcomes in service use and usability (*n* = 3) [26, 27], the ‘disease characteristics and sociodemographic characteristics’ was the most cited determinant with all three studies reporting it as an ambivalent factor (Supplementary table S4). Certain symptoms and conditions motivated the use of TM to obtain counseling and access to further care via telephone, such as pain and respiratory tract disorders [26]. Certain characteristics also influenced TM use, with women apparently being more disposed than men to use telephone consultations. No barrier or facilitator was cited more than once.

No study reported negative outcomes.

### TM effects on health-related and behavioural outcomes

Among the two different studies reporting on health-related and behavioural outcomes, one reported on the ability to cope with illness using a validated tool and one on general health status using a validated questionnaire [25, 30].

Only one study reported a positive outcome in health-related and behavioural outcomes [25]. This study only cited the ‘user habit or preferences’ as a determinant, reported as a barrier (Supplementary Table S4).

No study reported negative outcomes.

### Quality assessment

The quality of the included studies was overall very high (Table 3). Only six studies had one item or more with unknown risk of bias due to unavailable, insufficient or unclear information [19, 21, 22, 25, 28, 32]. Two studies had two items or more with unknown risk of bias [28, 32]. No study was at high risk on any of the methodological quality criteria.

**Table 3:** Mixed Methods Appraisal Tool (MMAT) scores of included studies

1^st^ author (Year), Country	Methodological Quality Criteria				
**Randomized Controlled Clinical Trial**					
	**Is randomization appropriately performed?**	**Are the groups comparable at baseline?**	**Are there complete outcome data?**	**Are outcome assessors blinded to the intervention provided?**	**Did the participants adhere to the assigned intervention?**
**Chae (2001) ** [27]**, Ireland**	✓	✓	✓	✓	✓
**Welch (2000) ** [19]**, USA**	✓	✓	✓	**Can’t tell**	✓
**Quantitative Non-Randomized Studies**					
	**Are the participants representative of the target population?**	**Are measurements appropriate regarding both the outcome and intervention (or exposure)?**	**Are there complete outcome data?**	**Are the confounders accounted for in the design and analysis?**	**During the study period, is the intervention administered (or exposure occurred) as intended?**
**Benaque (2020) ** [29]**, Spain**	✓	✓	✓	**X**	✓
**Jiwa (2005) ** [25]**, UK**	✓	✓	✓	**Can’t tell**	✓
**Khoong (2020) ** [24]**, USA**	✓	✓	✓	✓	✓
**Lam (2020) ** [23]**, 2020**	✓	✓	✓	✓	✓
**Van Houwelingen (2015) ** [20]**, Netherlands**	✓	✓	✓	✓	✓
**Quantitative Descriptive Studies**					
	**Is the sampling strategy relevant to address the research question?**	**Is the sample representative of the target population?**	**Are the measurements appropriate?**	**Is the risk of nonresponse bias low?**	**Is the statistical analysis appropriate to answer the research question?**
**Bujnowska-Fedak (2014) ** [28]**, Poland**	✓	**Can’t tell**	**Can’t tell**	✓	✓
**Jacome (2019) ** [26]**, Portugal**	✓	✓	✓	✓	✓
**Samples (2019) ** [22]**, USA**	**Can’t tell**	✓	✓	**X**	✓
**Townsend (2001) ** [21]**, USA**	✓	✓	✓	**Can’t tell**	✓
**Qualitative Studies**					
	**Is the qualitative approach appropriate to answer the research question?**	**Are the qualitative data collection methods adequate to address the research question?**	**Are the findings adequately derived from the data?**	**Is the interpretation of results sufficiently substantiated by data?**	**Is there coherence between qualitative data sources, collection, analysis and interpretation?**
**Blozik (2012) ** [35]**, Switzerland**	✓	✓	✓	✓	✓
**Foster (2001) ** [34]**, UK**	✓	✓	✓	✓	✓
**Franzosa (2021) ** [33]**, USA**	✓	✓	✓	✓	✓
**Kung (2016) ** [32]**, China**	✓	✓	**Can’t tell**	✓	**Can’t tell**
**Nymberg (2019) ** [31]**. Sweden**	✓	✓	✓	✓	✓
**Waterworth (2018) ** [30]**, New Zealand**	✓	✓	✓	✓	✓
**Mixed Methods Studies**					
	**Is there an adequate rationale for using a mixed methods design to address the research question?**	**Are the different components of the study effectively integrated to answer the research question?**	**Are the outputs of the integration of qualitative and quantitative components adequately interpreted?**	**Are divergences and inconsistencies between quantitative and qualitative results adequately addressed?**	**Do the different components of the study adhere to the quality criteria of each tradition of the methods involved?**
**Gabrielsson-Järhult (2021) ** [38]**, Sweden**	✓	✓	✓	✓	✓
**Macduff (2001) ** [37]**, Scotland**	**Can’t tell**	**Can’t tell**	**Can’t tell**	**Can’t tell**	**Can’t tell**
**Van Houwelingen (2018) ** [36]**, Netherlands**	✓	✓	✓	✓	✓

✓ = the paper adequately responds to the methodological quality criterion; X = the paper does not adequately respond to the methodological quality criterion; Can’t tell = the paper does not report appropriate information to answer ‘Yes’ or ‘No’ or reports unclear information related to the methodological quality criterion

## Discussion

This review included 21 studies of various designs to explore the effects of TM and the determinants of its use in older adults’ primary care. Both phone and videoconference technologies appeared equally reported. As older adults worldwide are still not using the internet and smartphones as much as their younger counterparts [50, 51], this equal distribution between higher and lower-tech options can be surprising. All primary and original studies reporting findings on TM in a context of primary care for older adults living in the community published before 2021, were included. We defined telemedicine as a synchronous telecommunication (phone, videoconference) in a primary care setting. Accordingly, we have decided not to set a publication start date to ensure that all synchronous TM interventions have been accounted for in our research.

According to our findings, TM also appears to lead to a generally positive experience among older adults. Previous reviews on TM among broader populations and not focused on family medicine similarly reported positive findings on patient satisfaction, despite highlighting methodological difficulties in their identified studies [6]. Our review described limited yet positive effects of TM on service use and on health-related outcomes. Other reviews reporting on the clinical effects of TM among an adult population provided encouraging findings, yet only targeted populations with specific conditions, such as diabetes or hypertension [52–54]. Additionally, these reviews mostly looked at clinical interventions relying heavily on monitoring [52], rather than TM as an alternative to in-person visits in primary care.

Lastly, the most commonly cited barrier from our findings pertained to ‘Technology skills and knowledge’ and the most commonly cited facilitator pertained to ‘Location/travel time’. Partially echoing our findings, Kruse et al. [55] further identified the decrease in travel time as a factor of satisfaction in their review on TM within the general population.

In hindsight, TM appears to be quickly moving from an emergent and innovative approach to a more mainstream type of care. Among the 21 studies included in this review, more than half (11/21, 52%) were published in the past five years and none were published before 2000. This already suggests a rapid increase in the interest on the topic. Undoubtedly, with the various applications of TM recently created in a state of emergency, the COVID-19 crisis will now spur the growth in this field towards an even more drastic expansion. Researchers worldwide are already starting to ask how much of the COVID era TM will remain and be definitely integrated in usual care [56].

Yet, there are still limited data targeting the specific population of older adults and the specific practice of primary care. From this review, most evidence were uncontrolled, non-randomized studies, with only two RCTs published on the topic, thus limiting the strength of recommendations [57].

Additionally, as this review illustrated, most reported data were concentrated around the experiences of primary care TM for older adults. Very few authors reported findings on the various outcomes of service use and on health-related outcomes. Furthermore, most reported determinants focused on the experiences of patients and healthcare providers, only briefly touching on organizations and technologies and mostly leaving aside the society and policy categories. Thus, based on this limited diversity in the reported variables, the relative diversity of the authors, the sheer number of publications and their recent date, the limited diversity or methods used and their types, and the high quality of the produced studies, the research field of primary care TM for older adults appears to be at an early to moderate maturity stage [58]. As this moderately new field begins its expansion, researchers will be expected to plan studies using additional study designs, such as RCTs, and to investigate further additional variables, notably service use and health-related outcomes. This addition of new data supporting the efficacy of TM in the primary care of older adults and validating the determinants of its use from various stakeholders’ perspectives, will then allow for more solid recommendations and a successful implementation in the near future [59].

The ‘primary care clinicians’ and ‘patients/caregivers’ categories of the human dimension and the ‘technology’ category of the system dimension all had determinants cited five times or more. From these, ‘comfort with patient communication’ (*n* = 5), ‘technology skills and knowledge’ (*n* = 7), ‘user habit/preferences’ (*n* = 6), ‘technology equipment’ (*n* = 6) and ‘reliability of technology’ (*n* = 5) were the most common barriers and ‘location/travel time’ was the most common facilitator.

The relationship older adults hold with the technology thus appears central to most barriers to TM use. Either through their possession of a specific device, their confidence in its ability to properly work, their own literacy and self-efficacy to effectively use it, or their preferences, several pitfalls await the implementation of TM among this population. However, the generational divide in technology use tend to narrow each year and more older adults are using internet now than ever [60]. Many registered an even more rapid increase in internet use with the coronavirus pandemic [61]. Nevertheless, the old age is not a homogeneous group and some older adults are still reluctant to adopt recent technology. Among them, researchers have identified ‘non-users’, ‘reluctant users’, and ‘apprehensive users’, each with different profiles but similar ages [62]. The implementation of primary care TM in older adults might then benefit from overcoming the barriers identified in this review, while targeting the specific groups in which they are most likely to occur.

The time savings associated with TM appears particularly appealing across studies. As caring for oneself and health-related activities can take up to 23 h per month for many older adults [63], the opportunity to limit transportation time may be highly meaningful for some.

### Strengths and limitations

This review is the first to provide specific conclusions on TM for older adults’ primary care. Other existing reviews on TM either targeted younger populations [6, 7], populations with specific conditions [52–54], or included specific interventions outside the scope of practice of primary care [64]. The methodology of this review relied on a rigorous and comprehensive systematic approach supported by a specialized librarian, a detailed framework [16] to structure data collection, and a validated tool for critical appraisal [18]. Its mixed method design also enlightened the significance of both qualitative and quantitative data to comprehend fully the complexities that underpin the use of TM with older adults. Yet, this review presents some limitations. Notably, the heterogeneity of outcome reported across studies prevented us to run any meta-analysis. Some studies only mentioned old age without specifying the exact age of their participants. Furthermore, not all studies reported medical conditions and comorbidities of their participants, preventing subgroup analysis. Finally, the completion of this systematic review during the COVID-19 pandemic may have impacted research results. Care provided in clinics was forced to tailor their practice to include TM as an option for remote and safe consultations. As a result, we believe further research on TM has been conducted, which may have increased and exaggerated the number of results.

### Impact on clinical practice

This review showed that TM might represent a suitable option for older adults, conditional to their clinical context, considering both healthcare professionals and patients’ specificities. In light of our findings, clinicians could thus direct their efforts to the following elements for an optimal use of TM by healthcare professionals:

- Ensure that clinicians or clinical teams feel able to maintain a clear communication with patients,
- Support the familiarization of clinicians with available communication technologies, to enhance their confidence in collecting comprehensive patients' information through these tools and facilitate interdisciplinary collaboration inside and outside clinical teams,
- Encourage leadership-driven TM initiatives and acknowledge or reward the contributions of peers or other groups and organisations in supporting these initiatives.

Considering patients’ specificities, clinicians could also assess how TM could align with their patients’ preferences by:

- Introducing tools adapted to their technology skills, supporting patients in their TM platform navigation as needed and providing assistance to foster self-efficacy,
- Promoting the advantages and benefits of selected TM, notably on travel and travel time, to further encourage its acceptability,
- Advocating for a greater accessibility of technological tools that could improve patients' health, to ensure the access of their patients to appropriate equipment.

## Conclusion

This review indicates that TM might be a promising option for older adults receiving primary healthcare. However, to foster TM use among this population, decision-makers should consider the clinical context and both the patient’s and the healthcare professional’s profiles. While more evidence is still needed on the efficacy of TM on various indicators for older adults seen in primary care, the time appears particularly ripe to provide such remote options, with a careful consideration of the determinants of its use.

## Supplementary Information

**Additional file 1.**

## Data Availability

All data generated or analysed during this study are included in this published article and its supplementary information files.
