# Supplementary material for: Telemedicine in the primary care of older adults: a systematic mixed studies review
Source: BMC Prim Care. 2023 Jul 20;24:152. doi: 10.1186/s12875-023-02085-7 (PMC10357882; doi:10.1186/s12875-023-02085-7)
Supplement: Supplementary file 1 — Additional file 1. [file 12875_2023_2085_MOESM1_ESM.docx]

### Supplementary text S1. Complete EMBASE search strategy

1  telemedicine.mp. (33037)

2  distance counsel*.mp. (18)

3  remote consult*.mp. (674)

4  telehealth.mp. (11717)

5  etherap*.mp. (19)

6  E-Counsel*.mp. (277)

7  health mobile.mp. (126)

8  mHealth.mp. (4757)

9  eHealth.mp. (4387)

10  telecare.mp. (938)

11  teleconsult*.mp. (10858)

12  ehealth*.mp. (4418)

13  teleguide*.mp. (7)

14  telemed*.mp. (33537)

15  Remote video*.mp. (187)

16  Video conferenc*.mp. (1449)

17  Video chat.mp. (120)

18  Video visit*.mp. (203)

19  telephone.mp. (93893)

20  Video consultat*.mp. (462)

21  tele-coach*.mp. (16)

22  Virtual provider*.mp. (8)

23  Virtual appointment*.mp. (28)

24  Virtual consult*.mp. (194)

25  Skype.mp. (793)

26  Telehealthcare.mp. (106)

27  E-health.mp. (4075)

28  Econsult*.mp. (228)

29  Health Informatic*.mp. (2886)

30  1 or 2 or 3 or 4 or 5 or 6 or 7 or 8 or 9 or 10 or 11 or 12 or 13 or 14 or 15 or 16

or 17 or 18 or 19 or 20 or 21 or 22 or 23 or 24 or 25 or 26 or 27 or 28 or 29 (146752)

31  Elderly.mp. (580606)

32  aging.mp. (634135)

33  Geriatric*.mp. (158008)

34  Older Adult*.mp. (107225)

35  Senior*.mp. (60434)

36  ageing.mp. (64194)

37  31 or 32 or 33 or 34 or 35 or 36 (1326974)

38  primary healthcare.mp. (7883)

39  Family Practice.mp. (10065)

40  Family physician*.mp. (19413)

41  General practice.mp. (102902)

42  Primary Care.mp. (162973)

43  Primary health care.mp. (80279)

44  38 or 39 or 40 or 41 or 42 or 43 (312072)

45  30 and 37 and 44 (1083)

### Supplementary table S2. Inclusion and exclusion criteria for study selection

| **Inclusion Criteria** | **Exclusion Criteria** |
| --- | --- |
| **Type of Study** | |
| Primary/original study reporting data (i.e. randomized controlled trials, non-randomized intervention studies, systematic literature or scoping reviews, observational studies, laboratory studies, qualitative studies, reports, thesis, protocol, abstract, case studies) in either English, French or Russian. | Other type of document or all studies with no original data (i.e. editorial, commentary, letter, erratum, blog publication, bulletin, non-systematic reviews, statements, full books…). If no methods, but author(s) “reviewed” articles, not a systematic review.  Exclude if the publication is only reporting the results of another study.  (i.e. blog post such as *“a recent study just found that…”*)  Exclude if no data (i.e. protocol, abstract with no results)  Exclude if published in another language than English, French or Russian. |
| **Population** | |
| Adults aged over 65 years old living in the community, caregivers (if cognitive or physical impairment require the presence of caregivers), or healthcare providers (providers involved in older adults’ care) with any disease and multimorbidity with any disease and multimorbidity, requiring treatment or follow-up in the primary care setting. | Population not focused exclusively on adults aged over 65 years old, living in the community.  Ex. nursing homes residents, children, caregivers of children with developmental delays…  Include if older adults are analysed separately or if the mean age of the sample is 65 years or over even if no age criteria was used for recruitment |
| **Intervention/Exposure** | |
| Telemedicine defined as synchronous telecommunication (phone, videoconference), either planned or unplanned, is a core component of the studied intervention. | Any other definition of telemedicine.  Ex. no direct interaction, forums only, email correspondence, text apps.  Include if the telecommunication happens either between the patient and the provider or between two providers  Include if telemedicine is not listed in the main objectives but specific results are presented |
| Telemedicine is provided by a primary care practice involving a family physician, a nurse, or any other healthcare allied professional of the clinic (e.g., social worker) in a rural or urban setting. | No involvement of a family physician or a nurse from the primary care practice. Provided by specialized services or facilities. Focused exclusively on the physical rehabilitation or exercises-delivery by the physiotherapist or kinesiologist.  Ex. psychiatry, geriatric day hospital, physiotherapy programs  Include if primary care and geriatric care collaboration.  Include if primary prevention provided by primary care physicians or nurse, even if involvement of a targeted condition.  Exclude if secondary or tertiary prevention. |
| Telemedicine in integrated in primary care. | Not integrated or aiming to be integrated in the overall primary care practice (targeting only some very specific moments of care) or only used for monitoring/compliance purposes  Ex. Focused exclusively on the after discharge from the hospital care  Ex. Focused on the medication adherence monitoring, or blood pressure monitoring, weight monitoring for cardiac failure patients  Ex. Only for recording physiological measures BUT include if allowing the patient to consult with their PCP |
| **Outcomes** | |
| Reporting outcomes related to experience, effects, determinants and other related outcomes (satisfaction, users’ experience, intention to use, expectations, frequency of emergency department visits)  Does not need to be the main reported outcome. | Not reporting on experience, effects, determinants and related outcomes.  Include at the title/abstract stage if any doubt, to read the full-text. |

**Exclusion codes:**

1. Duplication (n=289)
2. Type of study: Language other than English, French and Russian (n=4)
3. Type of study: not a primary/original study reporting data (n=43)
4. Population: Population not focused exclusively on adults aged over 65 years old, living in the community (n=93)
5. Telemedicine not defined exclusively as synchronous telecommunication (phone, videoconference), planned or unplanned (n=102)
6. No involvement of the family physician or a nurse from the primary care practice (specialized services) (n=33)
7. Not aiming to be integrated in the overall primary care practice (targeting only some very specific moments of care) or only used for monitoring/compliance purposes (n=21)
8. Not reporting on experience and related outcomes: Satisfaction, users’ experience, Intentions/Expectations (n=6)

| **Supplementary table S3. Reported Outcomes and determinants** | | | | | | | | | | | | | | | | | | | | | | | | | | | | | | | | | | | | | | | | |
| --- | --- | --- | --- | --- | --- | --- | --- | --- | --- | --- | --- | --- | --- | --- | --- | --- | --- | --- | --- | --- | --- | --- | --- | --- | --- | --- | --- | --- | --- | --- | --- | --- | --- | --- | --- | --- | --- | --- | --- | --- |
| **General care experience** | | | | | | | | | | | | | | | | | | | | | | | | | | | | | | | | | | | | | | | | |
| Studies | Type of TM | Outcome Direction (+/-) | 1.1 Comfort with workflow | 1.2 Comfort with patient communication | 1.3 Comfort with provider interaction | 1.4 Expertise with technology | 1.5 Education and training | 1.6 Resistance to change | 2.1 Disease/ sociodemographic characteristics | 2.2 Technology skills and knowledge | 2.3 User habits/preferences | 2.4 Location/travel time | 2.5 Patient awareness / support | 2.6 Technology equipment | 2.7 Medical cost (out-of-pocket) | 3.1 Leadership | 3.2 Change management | 3.3 Budget | 3.4 Workflow reengineering | 3.5 Organizational culture | 3.6 Hospital information systems | 3.7 Training and support | 4.1 Reliability of technology | 4.2 Storage | 4.3 System speed | 4.4 User interface / intended use /usability | 4.5 Data quality | 4.6 Transmission | 4.7 Interoperability | 4.8 Information security | 5.1 3rd party payers | 5.2 Technology infrastructure | 5.3 Reimbursement | 5.4 Insurance fee schedule | 5.5 Social norms and values / temporal trends | 6.1 Medical liability | 6.2 Practice certification and license | 6.3 Governmental authority | 6.4 Privacy and security rules | 6.5 Interface standards |
| Benaque et al.2020 | Phone  Video | Experience (+) |  |  |  |  |  |  |  |  | - | + |  | - |  |  |  |  |  |  |  |  |  |  |  | + - |  |  |  |  |  |  |  |  |  |  |  |  |  |  |
| Blozik et al. 2012 | Phone | Experience (D) |  |  |  |  |  |  |  |  |  |  |  |  |  |  |  |  |  |  |  |  |  |  |  |  |  |  |  |  |  |  |  |  |  |  |  |  |  |  |
| Bujnowska-Fedak et al. 2014 | Phone  Video | Readiness (D) |  |  |  |  |  |  | + - | - | + - | + - |  | - |  |  |  |  |  |  |  |  |  |  |  | + - |  |  |  |  |  |  |  |  |  |  |  |  |  |  |
| Chae et al. 2001 | Video | Satisfaction (+) |  | + |  |  |  |  | + - |  | + | + - |  |  |  |  |  |  | + |  |  |  |  |  |  | + - |  |  |  |  |  |  |  |  |  |  |  |  |  |  |
| Foster et al. 2001 | Phone | Experience (-) |  | - |  |  |  |  | + - | + | + - | + |  |  |  |  |  |  |  |  |  |  |  |  |  | - |  |  |  |  |  |  |  |  |  |  |  |  |  |  |
| Franzosa et al.2021 | Video | Experience (D) |  | - | + | + - |  |  | + - | - | - | + | - | - | - |  | + - |  | + |  |  |  | - |  |  | + - |  |  | + |  | + |  |  |  |  |  |  |  | + - |  |
| Gabrielsson-Järhult et al.  2021 | Video | Experience (+) |  |  |  |  |  |  |  | + |  |  |  |  |  |  |  |  |  |  |  |  |  |  |  |  |  |  |  |  |  |  |  |  |  |  |  |  |  |  |
| Jiwa et al. 2005 | Phone | Experience (+) |  |  |  |  |  |  |  |  | - |  |  |  |  |  |  |  |  |  |  |  |  |  |  |  |  |  |  |  |  |  |  |  |  |  |  |  |  |  |
| Khoong et al. 2020 | Video | Interest (+) |  |  |  |  |  |  |  | - | + - |  |  | - |  |  |  |  |  |  |  |  |  |  |  |  |  |  |  |  |  |  |  |  |  |  |  |  |  |  |
| Kung et al. 2016 | Video | Experience  Acceptability (D) |  |  |  |  |  |  | + - | - | + - | + | + | - | + - |  |  |  | + | - |  |  | - |  |  |  |  |  |  | +  - |  |  |  |  |  |  |  | + |  |  |
| Lam et al. 2020 | Phone | Readiness (D) |  |  |  |  |  |  | - | - |  |  |  |  |  |  |  |  |  |  |  |  |  |  |  |  |  |  |  |  |  |  |  |  |  |  |  |  |  |  |
| Macduff et al. 2001 | Video | Satisfaction (+) |  | - | + - |  | - | + | + - |  | + - | + - |  |  |  | + |  |  | + |  |  |  | +  - |  |  | + - | - |  |  |  |  |  |  |  |  |  |  |  |  |  |
| Nymberg et al. 2019 | Phone  Video | Experience (+) |  |  |  |  |  |  | + - | + - | + - | + |  |  | - |  |  |  | - | - |  |  | - |  |  | + - |  | + | - | +  - |  |  |  |  |  |  |  |  |  |  |
| Samples et al. 2019 | Video | Experience (+) | - | + - |  | + |  | + | + - | - | - | + - |  |  |  |  |  |  |  |  |  |  | - |  |  |  |  |  |  |  |  |  |  |  |  |  |  |  |  |  |
| Van Houwelingen et al. 2015 | Video | Readiness (D) |  |  | + | + - |  | + |  |  |  |  |  |  | + - |  |  |  |  |  |  |  |  |  |  |  |  |  |  |  |  |  |  |  |  |  |  |  |  |  |
|  |  | Attitude (+) |  |  |  |  |  |  |  |  |  |  |  |  |  |  |  |  |  |  |  |  |  |  |  |  |  |  |  |  |  |  |  |  |  |  |  |  |  |  |
| Van Houwelingen et al. 2018 | Video | Experience (D) |  |  |  | + |  |  | + - | - | - |  | + | - |  |  |  |  |  |  |  |  | - |  |  | + - |  |  |  |  |  |  |  |  |  |  |  |  |  |  |
| Waterworth et al.  2018 | Phone | Experience  (D) |  | - |  |  | + - |  | + - |  | +  - | + |  |  | +  - |  |  |  | - |  |  |  | +  - |  |  | + - |  |  |  |  |  |  |  |  |  |  |  |  |  |  |
| Welch et al.  2000 | Phone | Satisfaction (+) |  |  |  |  |  |  |  |  | +  - |  |  |  |  |  |  |  | + - |  |  |  |  |  |  |  |  |  |  |  |  |  |  |  |  |  |  |  |  |  |
| **Service use and usability** | | | | | | | | | | | | | | | | | | | | | | | | | | | | | | | | | | | | | | | | |
| Studies | Type of TM | Outcome Direction (+/-) | 1.1 Comfort with workflow | 1.2 Comfort with patient communication | 1.3 Comfort with provider interaction | 1.4 Expertise with technology | 1.5 Education and training | 1.6 Resistance to change | 2.1 Disease/ sociodemographic characteristics | 2.2 Technology skills and knowledge | 2.3 User habits/preferences | 2.4 Location/travel time | 2.5 Patient awareness / support | 2.6 Technology equipment | 2.7 Medical cost (out-of-pocket) | 3.1 Leadership | 3.2 Change management | 3.3 Budget | 3.4 Workflow reengineering | 3.5 Organizational culture | 3.6 Hospital information systems | 3.7 Training and support | 4.1 Reliability of technology | 4.2 Storage | 4.3 System speed | 4.4 User interface / intended use /usability | 4.5 Data quality | 4.6 Transmission | 4.7 Interoperability | 4.8 Information security | 5.1 3rd party payers | 5.2 Technology infrastructure | 5.3 Reimbursement | 5.4 Insurance fee schedule | 5.5 Social norms and values / temporal trends | 6.1 Medical liability | 6.2 Practice certification and license | 6.3 Governmental authority | 6.4 Privacy and security rules | 6.5 Interface standards |
| Benaque et al. 2020 | Phone  Video | TM Usability (D) |  |  |  |  |  |  |  |  | - | + |  | - |  |  |  |  |  |  |  |  |  |  |  | + - |  |  |  |  |  |  |  |  |  |  |  |  |  |  |
| Chae et al. 2001 | Video | Number of clinical visits (+) |  | + |  |  |  |  | + - |  | + | + - |  |  |  |  |  |  | + |  |  |  |  |  |  | + - |  |  |  |  |  |  |  |  |  |  |  |  |  |  |
| Franzosa et al. 2021 | Video | TM Usability  (D) |  | - | + | + - |  |  | + - | - | - | + | - | - | - |  | + - |  | + |  |  |  | - |  |  | + - |  |  | + |  | + |  |  |  |  |  |  |  | + - |  |
| Jacome et al.  2019 | Phone | TM Usability  (+) |  |  |  |  |  |  | + - |  | - |  |  |  |  |  |  |  |  |  |  |  |  |  |  |  |  |  |  |  |  |  |  |  |  |  |  |  |  |  |
| Townsend et al. 2001 | Phone | TM Usability  (D) |  | - |  |  |  |  | + - |  |  | + |  |  |  |  |  |  | - |  |  |  |  |  |  | + - |  |  |  |  |  |  |  |  |  |  |  |  |  |  |
| Van Houwelingen et al. 2018 | Video | TM Usability  (D) |  |  |  | + |  |  | + - | - | - |  | + | - |  |  |  |  |  |  |  |  | - |  |  | + - |  |  |  |  |  |  |  |  |  |  |  |  |  |  |
| Waterworth et al. 2018 | Phone | TM Usability  (D) |  | - |  |  | + - |  | + - |  | +  - | + |  |  | +  - |  |  |  | - |  |  |  | +  - |  |  | + - |  |  |  |  |  |  |  |  |  |  |  |  |  |  |
| Welch et al. 2000 | Phone | Number of Clinical visits  (N) |  |  |  |  |  |  |  |  | +  - |  |  |  |  |  |  |  | + - |  |  |  |  |  |  |  |  |  |  |  |  |  |  |  |  |  |  |  |  |  |
| **Healthcare-related and behavioural outcomes** | | | | | | | | | | | | | | | | | | | | | | | | | | | | | | | | | | | | | | | | |
| Studies | Type of TM | Outcome Direction (+/-) | 1.1 Comfort with workflow | 1.2 Comfort with patient communication | 1.3 Comfort with provider interaction | 1.4 Expertise with technology | 1.5 Education and training | 1.6 Resistance to change | 2.1 Disease/ sociodemographic characteristics | 2.2 Technology skills and knowledge | 2.3 User habits/preferences | 2.4 Location/travel time | 2.5 Patient awareness / support | 2.6 Technology equipment | 2.7 Medical cost (out-of-pocket) | 3.1 Leadership | 3.2 Change management | 3.3 Budget | 3.4 Workflow reengineering | 3.5 Organizational culture | 3.6 Hospital information systems | 3.7 Training and support | 4.1 Reliability of technology | 4.2 Storage | 4.3 System speed | 4.4 User interface / intended use /usability | 4.5 Data quality | 4.6 Transmission | 4.7 Interoperability | 4.8 Information security | 5.1 3rd party payers | 5.2 Technology infrastructure | 5.3 Reimbursement | 5.4 Insurance fee schedule | 5.5 Social norms and values / temporal trends | 6.1 Medical liability | 6.2 Practice certification and license | 6.3 Governmental authority | 6.4 Privacy and security rules | 6.5 Interface standards |
| Jiwa et al.  2005 | Phone | Ability to cope with illness (+) |  |  |  |  |  |  |  |  | - |  |  |  |  |  |  |  |  |  |  |  |  |  |  |  |  |  |  |  |  |  |  |  |  |  |  |  |  |  |
| Welch et al. 2000 | Phone | Health status  (N) |  |  |  |  |  |  |  |  | +  - |  |  |  |  |  |  |  | + - |  |  |  |  |  |  |  |  |  |  |  |  |  |  |  |  |  |  |  |  |  |

### Supplementary Table S4: Summary of barriers and facilitators identified in the included quantitative and qualitative studies, using Chang’s (2015) framework.

### Human Dimension

|  | | | | Barriers | Ambivalent | Facilitators |
| --- | --- | --- | --- | --- | --- | --- |
| Dimension | Determinants | | Relevant quote from the articles related to the framework dimension | Articles reporting the determinant as a barrier | Articles reporting the determinant as both a facilitator and a barrier | Articles reporting the determinant as a facilitator |
| **HUMAN DIMENSION** | **Healthcare Providers** | | | | | |
|  | 1.1 | Comfort with workflow | “Providers were concerned about negative impacts on their clinic flow” (Samples, 2019) | **n=1**  ^33^ | **n=0** | **n=0** |
|  | 1.2 | Comfort with patient communication | “Many expressed concerns about their needs being assessed over the telephone, including doubts about the ability of unknown doctors to make accurate diagnoses in these circumstances. […] ‘I don’t think it is advisable to talk to the doctor over the phone about what you are suffering with and what the symptoms are and so on. I think it is most important that a doctor sees you’. (Male, group 4.)” (Foster, 2001) | **n=5**  ^32,42,45,46,49^ | **n=1**  ^33^ | **n=1**  ^39^ |
|  | 1.3 | Comfort with provider interaction | “There was also the mutually perceived incidental benefit of opportunity for doctor-nurse communication, often carrying over beyond individual cases” (Macduff, 2001) | **n=0** | **n=1**  ^49^ | **n=2**  ^31,45^ |
|  | 1.4 | Expertise with technology | “Nurses with high technology experience (e.g., computers, microwaves, Skype, tablets) (n = 41) had a significantly lower negative affect score related to the use of home telehealth” (Van Houwelingen, 2018) | **n=0** | **n=2**  ^31,45^ | **n=2**  ^33,48^ |
|  | 1.5 | Education and training | “It was clear that this rather public learning process had been uncomfortable for some of the nurses involved” (Macduff, 2001) | **n=1**  ^49^ | **n=1**  ^42^ | **n=0** |
|  | 1.6 | Resistance to change | “Undoubtedly, this was related to the local team leader’s role in initiating the development, but it was clear that her colleagues also found the development interesting and worthwhile” (Macduff, 2001) | **n=0** | **n=0** | **n=3**  ^31,33,49^ |
|  | **Patients & Caregivers** | | | | | |
|  | 2.1 | Disease characteristics / sociodemographic characteristics | “Older adults 'described ‘The aging body as a barrier’ with impaired practical abilities such as trembling fingers or impaired vision or hearing” (Nymberg, 2019) | **n=1**  ^34^ | **n=13**  ^32,33,37-40,42-46,48,49^ | **n=0** |
|  | 2.2 | Technology skills and knowledge | “Older people believed that they were not able to accomplish certain technological tasks (low self-efficacy), but discovered that they actually were able to do so or could do so after a small suggestion on how to proceed” (Van Houwelingen, 2018) | **n=7**  ^33-35,40,44,45,48^ | **n=1**  ^43^ | **n=2**  ^46,50^ |
|  | 2.3 | User habits/preferences | “’I don’t feel this can work and doesn’t feel real to me. I prefer going to a doctor in a clinic, let the doctor see myself through his own eyes’“ (Kung, 2006) | **n=6**  ^33,36,37,41,45,48^ | **n=8**  ^30,35,40,42-44,46,49^ | **n=1**  ^39^ |
|  | 2.4 | Location/travel time | “One participant felt that e-consultation could break down geographical barriers that potentially reduce access to healthcare […]  'I don't have to waste my time coming in for check-ups'” (Kung, 2006) | **n=0** | **n=5**  ^33,38-40,49^ | **n=7**  ^32,41-46^ |
|  | 2.5 | Patient awareness / support | “Although some participants were against e-consultation because of difficulty with internet access, they would consider using the service with the help from family members and friends, as this participant described: ‘It [e-consultation] is only feasible if I can receive help from the younger ones, helping me to use the internet…’ [Fishermen, retired, female, age above 65]” (Kung, 2006) | **n=1**  ^45^ | **n=1**  ^44^ | **n=1**  ^48^ |
|  | 2.6 | Technology equipment | “26 scheduled visits were cancelled due to lack of patients’ telecommunications devices” (Benaque, 2020) | **n=6**  ^35,40,41,44,45,48^ | **n=0** | **n=0** |
|  | 2.7 | Medical cost (out-of-pocket) | “Relying on paid caregivers often meant using the aides’ own phone and data plan. As one provider asked, ‘is someone reimbursing [the aide] for that [data]? Is there Wi-Fi in the home? Do they [the aide] even have a smartphone?’ (CD, Practice 6)” (Franzosa, 2021) | **n=2**  ^43,45^ | **n=3**  ^31,42,44^ | **n=0** |

In the human dimension, determinants pertained to both categories of primary care clinicians and patients/caregivers. In the ‘primary care clinicians’ category, the comfort with patient communication was the most cited determinant, reported as a barrier by 5 studies,^32,42,45,46,49^ as a facilitator by one study,^39^ and as a more ambivalent factor by one other study.^33^ It was therefore also the most cited barrier. Most concerns were about the loss of non-verbal cues. The most cited facilitator was the resistance to change, here expressed as an acceptance of that change by all three studies reporting on that determinant.^31,33,49^ Many mentioned a strong interest, optimism or even leadership in implementing the technological shift toward TM. The comfort with workflow, the comfort with other providers’ communication, the expertise with technology and the education and training were also reported as determinants of TM use. Overall, there were seven mentions of barriers and eight of facilitators.

In the ‘patients/caregivers’ category, the user habits and preferences was the most cited determinant, reported as a barrier by six studies,^33,36,37,41,45,48^ as a facilitator by one study^39^ and as a more ambivalent or descriptive factor by eight studies.^30,35,40,42-44,46,49^ Many older adults expressed how they preferred face-to-face interactions and remain within familiar territory, while some mentioned positive experiences with the technology, on which they were ready to build to learn about TM. The most cited barrier was the technology skills of the patients and their caregivers, reported as a barrier by seven studies,^33-35,40,44,45,48^ as a facilitator by two studies^46,50^ and as more ambivalent by one study.^43^ Many mentioned distrust, inexperience, unreadiness or lack of self-efficacy with the technology, with some older adults even claiming they were ‘digital illiterate’. The most cited facilitator was the location and travel time, reported as a facilitator by seven studies^32,41-46^ and as a more ambivalent or descriptive factor by five studies.^33,38-40,49^ Most saw in TM an increased accessibility, particularly for rural areas, and convenient time saving from both sides. Interestingly, technology equipment was the only determinant exclusively cited a barrier. Six studies^35,40,41,44,45,48^ reported on either lack of computer or lack of internet or data access. The specific disease/sociodemographic characteristics, the patient awareness and support with TM and out-of-pocket costs were also reported as determinants of TM use. Overall, there were 23 mentions of barriers and 12 of facilitators.

### System Dimension

|  | | | | Barriers | Ambivalent | Facilitators |
| --- | --- | --- | --- | --- | --- | --- |
| Dimension | Determinants | | Relevant quote from the articles related to the framework dimension | Articles reporting the determinant as a barrier | Articles reporting the determinant as both a facilitator and a barrier | Articles reporting the determinant as a facilitator |
| **SYSTEM DIMENSION** | **Organization** | | | | | |
|  | 3.1 | Leadership | “Undoubtedly, this was related to the local team leader’s role in initiating the development” (Macduff, 2001) | **n=0** | **n=0** | **n=1**  ^49^ |
|  | 3.2 | Change management | “While participants generally expressed pride in how quickly they and their patients adapted, they also described limitations […] including the need to rapidly consent patients, set up patient portal accounts, and learn a new system quickly” (Franzosa, 2021) | **n=0** | **n=1**  ^45^ | **n=0** |
|  | 3.3 | Budget | N/A | **n=0** | **n=0** | **n=0** |
|  | 3.4 | Workflow reengineering | “Instead of providing a way to maintain contact with patients without requiring them to appear in clinic frequently, telephone appointments became simply an additional service” (Welch, 2000) | **n=3**  ^32,42,43^ | **n=1**  ^30^ | **n=4**  ^39,44,45,49^ |
|  | 3.5 | Organizational culture | “They also had thoughts about differences between the organizations. One of the participants wondered why short text message reminders are common in the dental care but not in primary care” (Nymberg, 2019) | **n=2**  ^43,44^ | **n=0** | **n=0** |
|  | 3.6 | Hospital information systems | N/A | **n=0** | **n=0** | **n=0** |
|  | 3.7 | Training and support | N/A | **n=0** | **n=0** | **n=0** |
|  | **Technology** | | | | | |
|  | 4.1 | Reliability of technology | “Some participants demanded high internet stability for the service, as they felt it would be useless if the technology itself was unreliable: ‘If the computer system is slow then it [e-consultation] isn’t helpful. It will take longer if the computer system constantly breaks down and need to spend hours to recover.’” (Kung, 2006) | **n=5**  ^33,43-45,48^ | **n=2**  ^42,49^ | **n=0** |
|  | 4.2 | Storage | N/A | **n=0** | **n=0** | **n=0** |
|  | 4.3 | System speed | N/A | **n=0** | **n=0** | **n=0** |
|  | 4.4 | User interface / intended use /usability | “Overall, providers noted the diversity of options (institutional platforms, other HIPAA-compliant commercial platforms, and consumer platforms) allowed greater access to patients than would otherwise have been possible. Providers appreciated the ease of texting images of a skin condition or meeting quickly by FaceTime and hoped this flexibility could continue as privacy rules were enforced again. ‘We just did whatever we really felt was needed for that patient, and it’s going to be spoiled going back to the regular [institutional platform] way’, noted one social worker” (Franzosa, 2021) | **n=1**  ^46^ | **n=9**  ^32,39-43,45,48,49^ | **n=0** |
|  | 4.5 | Data quality | “GPs [General Practitioners] reported some sound issues, difficulties seeing rashes and skin problems.”(Macduff, 2001) | **n=1**  ^49^ | **n=0** | **n=0** |
|  | 4.6 | Transmission | “Having digital access to information about the medication was described as another potential advantage” (Nymberg, 2019) | **n=0** | **n=0** | **n=1**  ^43^ |
|  | 4.7 | Interoperability | “The main issue that the participants talked about was that there was ‘Poor communication between health care organizations’ IT systems’. As no organization was fully updated with all the information, the participants expressed ‘disappointment over poor IT systems’” (Nymberg, 2019) | **n=1**  ^43^ | **n=0** | **n=1**  ^45^ |
|  | 4.8 | Information security | ““I think e-consultation opens up an opportunity for criminal activities if safety measures [online security] are not taken.’ [Police, retired, male, age above 65]” (Kung, 2006) | **n=0** | **n=2**  ^43,44^ | **n=0** |

In the system dimension, determinants pertained to both categories of organization and technology. In the ‘organization’ category, the workflow reengineering was the most cited determinant, reported as a barrier by three studies,^32,42,43^ as a facilitator by four studies^39,44,45,49^ and as a more ambivalent factor by one study.^30^ Many saw a benefit in TM, notably in triaging patients toward more relevant care and some even reported reduced caseloads for physicians as a result. It was also the most cited barrier, as for some, TM only resulted in an additional service to provide, thereby leading to additional costs. TM workflow reengineering was the most cited facilitator as well. The leadership of the organization, its change management and the organizational culture were also reported as determinants of TM use. Overall, there were five mentions of barriers and five mentions of facilitators.

In the ‘technology’ category, the user interface/intended use/usability was the most cited determinant, reported as a barrier by one study^46^ and as a more ambivalent or descriptive factor by nine studies.^32,39-43,45,48,49^ If the recorded messages were not easy to understand, the use of the platform was compromised. Most study participants expressed how much they cared about that aspect, describing various experiences, thoughts and worries. The most cited barrier was the reliability of the technology, reported as a barrier by five studies^33,43-45,48^ and as a more ambivalent factor by two studies.^42,49^ The quality and speed of the technology used needed to allow for stable and seamless service, otherwise patients often expressed dissatisfaction or disappointment. The most cited facilitators were about the transmission of information, reported as a facilitator by one study,^43^ and the interoperability of the technology, reported as a barrier by one study^43^ and as a facilitator by one other study.^45^ Easy access to patient information and the possibility to share and collaborate with other providers were greatly appreciated. Overall, there were eight mentions of barriers and two mentions of facilitators.

### Environmental Dimension

|  | | | | Barriers | Ambivalent | Facilitators |
| --- | --- | --- | --- | --- | --- | --- |
| Dimension | Determinants | | Relevant quote from the articles related to the framework dimension | Articles reporting the determinant as a barrier | Articles reporting the determinant as both a facilitator and a barrier | Articles reporting the determinant as a facilitator |
| **ENVIRONMENT DIMENSION** | **Society** | | | | | |
|  | 5.1 | 3rd party payers | “The accelerated pace of change driven by the pandemic and resulting changes in regulation and reimbursement have also allowed for rapid HBPC practice innovations that would not otherwise have been possible.” (Franzosa, 2021) | **n=0** | **n=0** | **n=1**  ^45^ |
|  | 5.2 | Technology infrastructure | N/A | **n=0** | **n=0** | **n=0** |
|  | 5.3 | Reimbursement | N/A | **n=0** | **n=0** | **n=0** |
|  | 5.4 | Insurance fee schedule | N/A | **n=0** | **n=0** | **n=0** |
|  | 5.5 | Social norms and values / temporal trends | N/A | **n=0** | **n=0** | **n=0** |
|  | **Rules/Policy** | | | | | |
|  | 6.1 | Medical liability | N/A | **n=0** | **n=0** | **n=0** |
|  | 6.2 | Practice certification and license | N/A | **n=0** | **n=0** | **n=0** |
|  | 6.3 | Governmental authority | “Governmental regulations in the establishment and running of e-consultation services would enhance participants’ trust in the service. ’As long as the government is at the back of the service [e-consultation], I would then have confidence in it.’ [Domestic helper, retired, female, age 40-65]” (Kung, 2006) | **n=0** | **n=0** | **n=1**  ^44^ |
|  | 6.4 | Privacy and security rules | “Providers appreciated the ease of texting images of a skin condition or meeting quickly by FaceTime and hoped this flexibility could continue as privacy rules were enforced again. ‘We just did whatever we really felt was needed for that patient, and it’s going to be spoiled going back to the regular [institutional platform] way’, noted one social worker” (Franzosa, 2021) | **n=0** | **n=1**  ^45^ | **n=0** |
|  | 6.5 | Interface standards | N/A | **n=0** | **n=0** | **n=0** |

In the environmental dimension, determinants pertained to both categories of society and rules/policy. In the ‘society’ category, third party payers and social norms and values or temporal trends were the most cited determinants, both reported as facilitators by one study respectively.^38,45^ They were therefore also the most cited facilitators. Indeed, the recent pandemic accelerated the development of regulations and reimbursements for TM consultations.^45^ In addition, older patients demonstrated a preference toward using TM over in-person consultations following temporal trends. Overall, there were two mentions of facilitators but no mention of barriers.

In the ‘rules/policy’ category, government authority and privacy and security rules were the most cited determinants, reported as a facilitator by one study^44^ and as a more ambivalent factor by one study^45^ respectively. Government authority was therefore also the most cited facilitator. Older adults anchored their confidence and trust in TM in government involvement in its regulations. In addition, healthcare professionals who used TM with older patients during the pandemic were grateful for its rapidity and flexibility during those difficult times but were wary of losing this ease of use when the health crisis end and the exceptions to privacy rules are waived. Overall, there was only one mention of facilitators and no mention of barriers.
